# Feasibility of Elective Nodal Irradiation (ENI) and Involved Field Irradiation (IFI) in Radiotherapy for the Elderly Patients (Aged ≥ 70 Years) with Esophageal Squamous Cell Cancer: A Retrospective Analysis from a Single Institute

**DOI:** 10.1371/journal.pone.0143007

**Published:** 2015-12-04

**Authors:** Wang Jing, Hui Zhu, Hongbo Guo, Yan Zhang, Fang Shi, Anqin Han, Minghuan Li, Li Kong, Jinming Yu

**Affiliations:** 1 Weifang Medical University, Weifang, Shandong Province, China; 2 Department of Radiation Oncology, Shandong Cancer Hospital and Institute, Jinan, Shandong Province, China; 3 Department of Thoracic Surgery, Shandong Cancer Hospital and Institute, Jinan, Shandong Province, China; University of California Davis, UNITED STATES

## Abstract

**Purpose:**

We conducted a retrospective analysis to assess the feasibility of involved field irradiation (IFI) in elderly patients with esophageal squamous cell cancer (ESCC).

**Materials and Methods:**

We performed a retrospective review of the records of elderly patients (≥ 70 years) with unresectable ESCC and no distant metastases who received treatment with radiotherapy between January 2009 and March 2013. According to the irradiation volume, patients were allocated into either the elective nodal irradiation (ENI) group or the IFI group. Overall survival (OS), progression-free survival (PFS) and treatment-related toxicities were compared between the two groups.

**Results:**

A total of 137 patients were enrolled. Fifty-four patients (39.4%) were allocated to the ENI group and 83 patients (60.6%) to the IFI group, the median doses in the two groups were 60 Gy and 59.4 Gy, respectively. For the entire group, the median survival time (MST) and PFS were 16 months and 12 months, respectively. The median PFS and 3-year PFS rate in the ENI group were 13 months and 20.6%, compared to 11 months and 21.0% in the IFI groups (*p* = 0.61). The MST and 3-year OS rate in the ENI and IFI groups were 17 months and 26.4% and 15.5 months and 21.7%, respectively (*p* = 0.25). The rate of grade ≥ 3 acute irradiation esophagitis in the ENI group was significantly higher than that in the IFI group (18.5% vs. 6.0%; *p* = 0.027). Other grade ≥ 3 treatment-related toxicities did not significantly differ between the two groups.

**Conclusions:**

IFI resulted in decreased irradiation toxicities without sacrificing OS in elderly patients with ESCC.

## Introduction

Esophageal cancer (EC) is the fifth leading cause of cancer-related deaths globally in men and the eighth in women. An estimated 482,300 new EC cases occurred in 2008 worldwide [1]. With the aging of the population, it is estimated that approximately 70% of all cancers will be diagnosed in the elderly by 2030, and cancer has become the leading cause of death among the elderly in the United States [2, 3]. However, elderly patients are underrepresented in clinical trials, and specific treatment recommendations for this group are often lacking. With the extension of life expectancy and the need for high quality of life, evidence-based data are essential to guide the treatment of these patients. Notwithstanding numerous disadvantages from which these patients may suffer, such as physiologic status and comorbidities, aggressive treatment should not be avoided in the heterogeneous elderly population. Furthermore, the toxicities and side effects induced by treatment and the benefits of survival should be well balanced.

Radiotherapy combined with chemotherapy is the definitive treatment strategy for patients with unresectable EC and those who refuse surgery. Elective nodal irradiation (ENI) is recommended for patients with EC because it can improve local regional control (LRC) and prolong overall survival (OS), but this modality is usually accompanied by serious treatment-related toxicities such as grade ≥ 3 irradiation esophagitis (RE) [4–7]. Under the supposition that involved field irradiation (IFI) could decrease the treatment-related toxicities because of the smaller target volume than that of ENI, the role of IFI in locally advanced non-small cell lung cancer (LANSCLC) and EC has been evaluated in recent years. Now that IFI has been demonstrated to improve outcomes without increasing toxicity in LANSCLC, it has become the standard irradiation target volume [8].

Although a few studies affirmed that the OS, progression-free survival (PFS), and LRC of IFI were similar to the results of ENI acquired in the Irradiation Therapy Oncology Group (RTOG) trials, and with lower toxicities observed for IFI than those found for ENI [4, 9–12], high-grade evidence for the use of IFI in EC is in shortage, particularly for elderly patients. Therefore, we performed a retrospective study to investigate the effect and toxicities of IFI in elderly patients with unresectable esophageal squamous cell cancer (ESCC) and compared the outcomes to those achieved with ENI in the same setting.

## Materials and Methods

### Patients

We retrospectively reviewed the records of patients with histologically-proven ESCC treated in our cancer center between January 2009 and March 2013. According to the criteria of the American Joint Committee on Cancer (AJCC) 7^th^ edition (2010), all contrast-enhanced chest computed tomography (CT) (including localizable CT) and positron emission tomography (PET)/-CT imaging data were reviewed by at least two experienced radiologists. N0 was defined as no regional lymph nodes (LNs) metastases, while N+ was defined as positive regional LNs. Positive supraclavicular LNs, left gastric arterial and celiac axis nodes as well as regional LNs were included. According to the difference in the irradiation field, all patients treated with radiotherapy were divided into either the ENI group or the IFI group, with chemotherapy or not. Patients were excluded if they had carcinoma in any other site or had a history of prior treatment. All study participants provided informed consent. Patient information was anonymized and de-identified prior to analysis. The study was approved by the institutional review board and ethics committee at the Shandong Cancer Hospital and Institute.

### Radiotherapy

Radiation therapy was to be delivered with three-dimensional conformal radiotherapy or Intensity Modulated Irradiation Therapy. Treatment plans were generated with a three-dimensional planning system (ADAC-Pinnacle 3, version 5.0). Patients were treated 5 days per week at 1.8–2.0 Gy, with a total dose of 50–68.4 Gy. For IFI, the gross tumor volume (GTV) was defined as any visible esophageal lesions (GTVt) shown on CT, barium esophagography, localizable CT or diagnostic CT images and PET/CT scans, plus any involved LNs (GTVnd). The criterion for LN involvement was at least one of the following: diameter of the short axis ≥ 10 mm, diameter of the long axis ≥ 15mm, or high standardized uptake values on PET/CT images. The presence of extra-nodal tumor extension and peripheral enhanced LNs helped us to determine metastatic status. The clinical target volume (CTV) consisted of CTVt and CTVnd. The CTVt was defined as the GTVt plus a 2.0–4.0 cm margin superior and inferior to the primary tumor and a lateral margin of 0.8–1.0 cm. CTVnd was defined as the GTVnd plus a 0.5–1.0 cm radial margin. The planning target volume (PTV) was defined as the CTV plus a 0.5–1.0 cm radial margin. For ENI, the GTV was defined in the same way as that for IFI. CTV was defined as GTV plus a 2.0–5.0 cm craniocaudal margin with a 0.8–1.0 cm lateral margin and the areas at risk for elective nodal regions (such as lower cervical, periesophageal, mediastinal and perigastric LNs). Elective treatment of nodal regions depended upon the location of the primary tumor. For example, the supraclavicular nodes and the celiac nodes were included for the upper thoracic esophagus and the lower esophagus, respectively. The definition of PTV was the same as that for IFI.

For ENI, all LN stations received a minimum irradiation dose of 40 Gy. A booster dose was further administered to the primary tumor and the involved LN up to a total dose of 50–68.4 Gy.

### Chemotherapy

In the whole group, 65 (47.4%) patients underwent chemotherapy, including induction chemotherapy (16.9%), concurrent chemotherapy (62.3%) and sequential chemotherapy (16.9%). Among the patients who underwent chemotherapy, 27 (41.5%) were in the ENI group and 38 (58.5%) were in the IFI group. For induction chemotherapy, irradiation therapy was started 1 week after chemotherapy was finished. Concurrent chemotherapy began from the first day of irradiation. Sequential chemotherapy was started 1 week after the radiotherapy was completed. Typically, patients received 2 cycles of platinum-based chemotherapy with combined 5-fluorouracil and a taxane (docetaxel or paclitaxel).

### Toxicity and Endpoints

Treatment-induced toxicities, including Leukopenia, anemia, thrombocytopenia, esophagitis and pneumonitis, were assessed using the RTOG grading criteria. The observation started from the date of treatment to the date of death or the last follow-up.

The primary endpoint was OS and the secondary endpoint was PFS. OS was measured from the first day of treatment to the date of death from any cause or the last known date that the patient was alive. PFS was defined as the duration from the date of treatment to the date of failure, either the date of death from any cause or the date of the last known follow-up.

### Follow-Up

A review of the radiography findings (CT, esophagography) was performed to assess the response after chemotherapy, and at the end of treatment. The response was evaluated according to the Response Evaluation Criteria in Solid Tumors version 1.0. Patients were followed up every 3 months for the first year, and then every 6 months for the following 3 years, and annually thereafter. The follow-up evaluations consisted of history, physical examination, and radiologic examinations, including esophagography, and chest and abdominal CT. Other necessary examinations were conducted according to the clinical situation.

### Statistical Analysis

Comparisons of patient characteristics and toxicities were performed with Chi-square (and Fisher’s exact) test. OS and PFS were calculated using the Kaplan-Meier method, and the log-rank test was employed to evaluate the difference in survival curves between the ENI and IFI groups. Multivariate analysis for OS was performed using Cox regression and a backward-forward stepwise method was selected. Two-sided *p* values < 0.05 were considered statistically significant.

## Results

### Patients

Between January 2009 and March 2013, 513 patients were diagnosed with ESCC and accepted treatment with radiotherapy in our cancer center, and only 164 of these patients were 70 years of age or older. Of those 164 patients, 27 patients (16.5%) were lost to follow-up and ultimately 137 patients were enrolled in the current study. Among this group, 54 patients (39.4%) received ENI and 83 patients (60.6%) received IFI. The median follow-up period for all patients was 16.4 months (range, 3–66months). At the time of the last follow-up, 30 patients (21.9%) were still alive. The median age of all patients was 75 years (range 70–88 years). The baseline characteristics of the patients in the two groups are detailed in Table 1.

**Table 1:** Clinical features of esophageal cancer patients treated with elective nodal irradiation or involved-field irradiation.

Characteristic	N	ENI (%)	IFI (%)	*p* value
**Sex**				
Male	80	33 (61.1)	47 (56.6)	0.72
Female	57	21 (38.9)	36 (43.4)	
Median age (range)		75 (70–88)		
**T stage**				
T1-3	102	45 (83.3)	57 (68.7)	0.29
T4	35	9 (16.7)	26 (31.3)	
**LN status**				
N0	33	15 (27.8)	18 (21.7)	0.98
N+	104	39 (72.2)	65 (78.3)	
**Location**				
Cervical	11	6 (11.1%)	5 (6.0%)	0.34
Thoracic	126	48 (88.9%)	78 (94.0%)	
**Tumor length (cm)**				
Median (range)		5.0 (1.5–10.0)	5.0 (2.2–14.0)	
≤ 5cm	74	30	44	0.86
> 5cm	63	24	39	
**RT dose (Gy)**				
Median (range)		60 (50–68.4)	59.4 (50–66)	
≥ 60	67	32 (59.3)	35 (42.2)	0.06
< 60	60	22 (40.7)	48 (57.8)	
**KPS score**				
≥ 80	118	48 (88.9)	70 (84.3)	0.61
< 80	19	6 (11.1)	13 (15.7)	
**Smoking status**				
Yes	72	30 (55.6)	42 (50.6)	0.60
No	65	24 (44.4)	41 (49.4)	
**Chemotherapy**	65	27 (41.5)	38 (58.5)	
Induced chemotherapy	25	8 (29.6)	17 (44.7)	0.39
Concurrent chemotherapy	35	16 (59.3)	19 (50)	
Sequential chemotherapy	5	3 (11.1)	2 (5.3)	

ENI, elective nodal irradiation; IFI, involved field irradiation; LN, lymph node; RT, radiotherapy; KPS, Karnofsky performance status

### Survival

**Fig 1:**
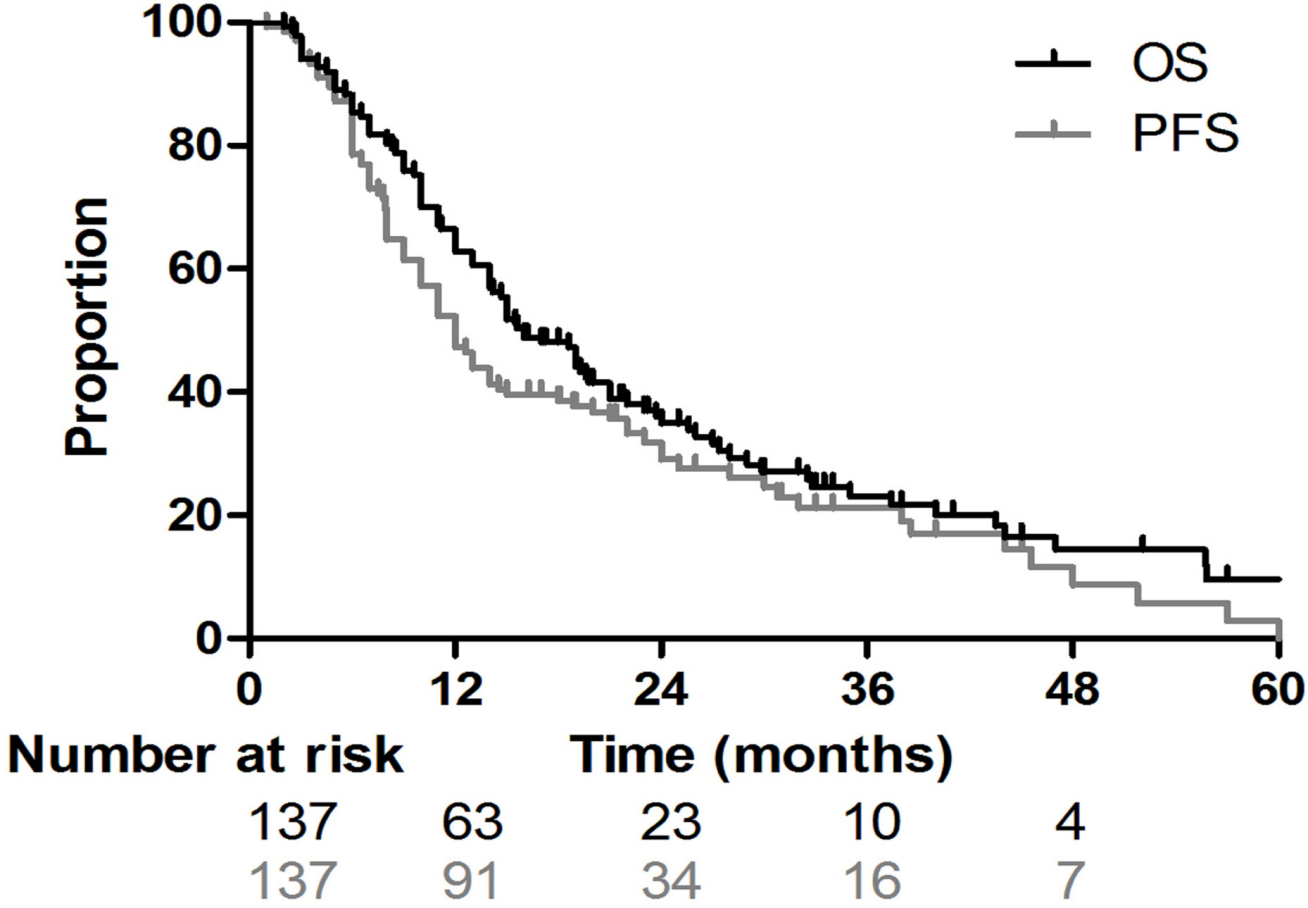
Overall survival (OS) and progression-free survival (PFS) curves illustrate the survival of all patients. For the whole cohort of patients with esophageal squamous cell cancer who underwent radiotherapy, the median OS and PFS were 16 months and 12 months, respectively. The 1-, 2-, and 3-year OS and PFS rates for the entire population were 62.8%, 35.0%, and 23.2%, and 47.4%, 29.1%, and 21.2%, respectively.

**Fig 2:**
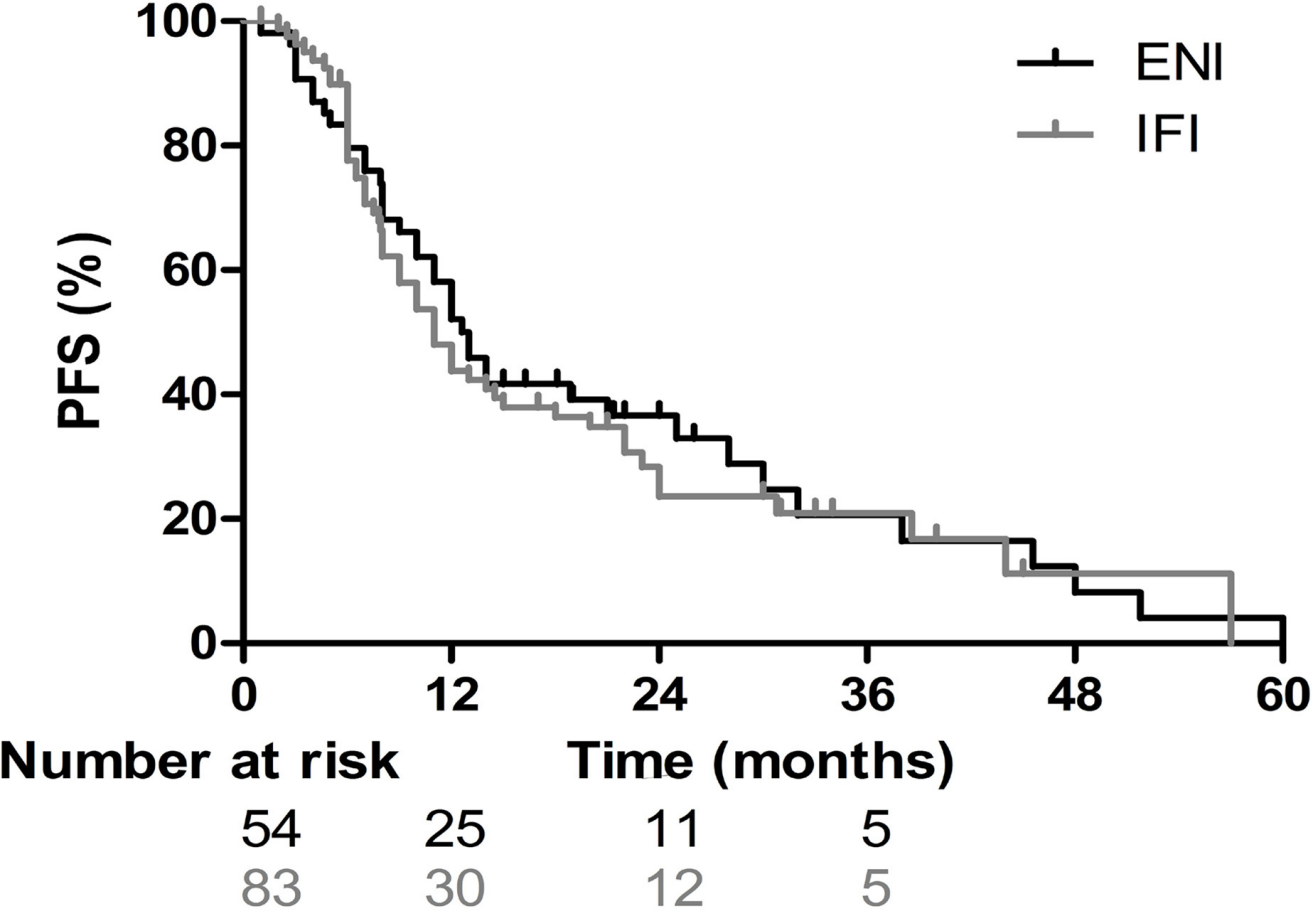
Progression-free survival (PFS) curves illustrate the survival of patients receiving elective nodal irradiation (ENI) and involved-field irradiation (IFI). The median PFS for patients in the IFI and ENI groups was 11 months and 13 months, respectively; the 1-, 2-, and 3-year PFS rates for patients between the IFI and ENI groups were 43.8%, 23.6%, and 21.0%, and 52.1%, 36.6% and 20.6%, respectively (*p* = 0.61).

**Fig 3:**
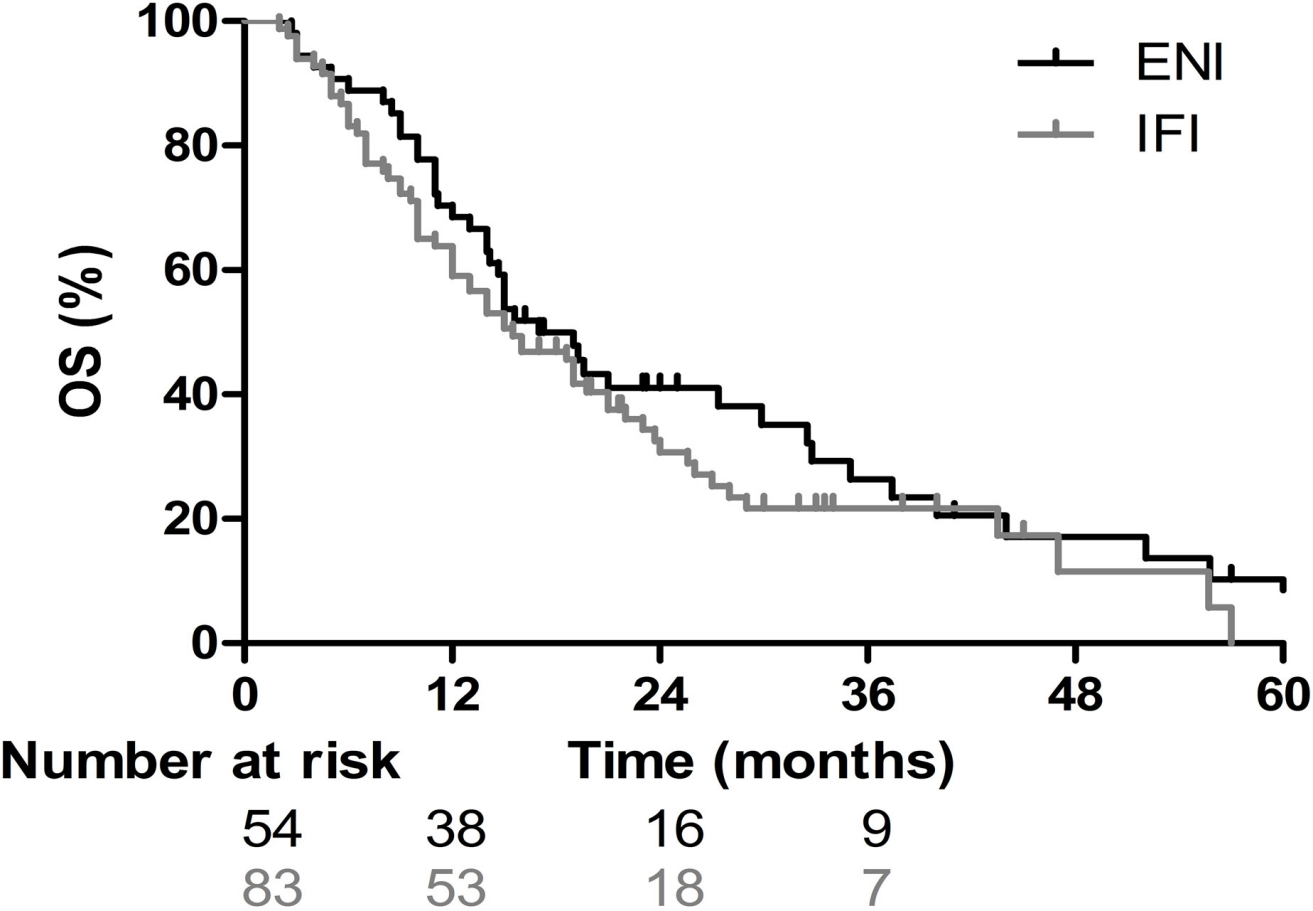
Overall survival (OS) curves illustrate the survival of patients receiving elective nodal irradiation (ENI) and involved-field irradiation (IFI). The median survival times of the patients in the IFI and ENI groups were 15.5 months and 17 months, respectively, and the 1-, 2- and 3-year survival rates were 59.0%, 30.7%, and 21.7%, and 68.5%, 41.0%, and 26.4%, respectively (*p* = 0.25).

For the entire group, the median OS and PFS were 16 months and 12 months, respectively. The 1-, 2-, and 3-year OS and PFS rates for the entire population were 62.8%, 35.0%, and 23.2%, and 47.4%, 29.1%, and 21.2%, respectively (Fig 1). For the 83 patients in the IFI group, the median PFS and the 1-, 2-, and 3-year PFS rates were 11 months, 43.8%, 23.6% and 21.0%, respectively; for the 54 patients in the ENI group, they were 13 months, 52.1%, 36.6% and 20.6%, respectively (*p* = 0.61; Fig 2). The MSTs of the patients in the IFI and ENI groups were 15.5 months and 17 months, respectively, and the 1-, 2- and 3-year survival rates were 59.0%, 30.7%, and 21.7% and 68.5%, 41.0%, and 26.4%, respectively (*p* = 0.25, Fig 3).

### Prognostic Factors

Patient characteristics were evaluated to determine their prognostic value for OS (Table 2). Univariate analysis revealed that irradiation field, sex, LN status, location, tumor length, radiotherapy dose, KPS, smoking status and treatment strategy were not associated with survival; however, T stage (*p* = 0.033) was a significant prognostic factors for survival. Multivariate analysis revealed that T stage (hazard ratio [HR] = 1.60, *p* = 0.037) was an independent prognostic factors for OS.

**Table 2:** Univariate and multivariate analysis of the effect of prognostic factors on OS in patients with esophageal cancer.

Factors	Univariate analysis					Multivariate analysis		
	1-y OS (%)	3-y OS (%)	5-y OS (%)	χ2	*p*	HR	95% CI	*p*
Male	55.0	20.8	12.5	1.467	0.226			
Female	73.7	26.9	0					
**Tumor stage**								
T1-3	64.7	28.4	7.6	4.529	0.033	1.60	1.028–2.495	0.037
T4	57.1	0	0					
N0	60.6	32.8	10.9	1.245	0.264			
N+	63.5	20.2	4.7					
Cervical	90.9	23.4	11.7	2.252	0.133			
Thoracic	60.3	23.2	5.1					
≤ 5	64.9	31.7	5.6	2.209	0.154			
> 5	60.3	16.0	5.7					
≥ 60	65.7	27.7	5.3	0.293	0.588			
< 60	60.0	19.0	6.3					
≥ 80	60.0	19.0	6.3	0.293	0.588			
< 80	65.7	27.7	5.3					
Yes	69.4	22.8	3.5	0.066	0.797			
No	55.4	22.9	9.2					
RT alone	63.9	21.2	4.7	0.422	0.936			
CCRT	57.1	32.1	6.9					
C+RT	64.0	17.5	8.8					
RT+C	80.0	0	0					
ENI	68.5	26.4	10.3	1.319	0.251			
IFI	59.0	21.7	0					

### Toxicity

Treatment-induced toxicities for all patients are detailed in Table 3. Leukopenia, esophagitis and pneumonia were the major toxicities. No significant difference was found between ENI patients and IFI patients who experienced grade ≥ 3 treatment-related hematologic toxicities. The rate of grade ≥ 3 acute RE in the ENI group (10/54, 18.5%) was significantly higher than that in the IFI group (5/83, 6.0%), with a *p* value of 0.027. The incidence of grade ≥ 3 acute irradiation pneumonitis (RP) was 7.4% in the ENI group (4/54) and 12.0% in the IFI group (10/83), but the difference was not significant (*p* = 0.40). However, 10 patients in the IFI group and 2 patients in the ENI group died from RP.

**Table 3:** Incidence of toxicity in the patients with esophageal cancer treated with elective nodal irradiation (ENI) or involved-field irradiation (IFI).

Toxicity/Grade	No. of Patients (%)		*p*
	ENI (n = 54)	IFI (n = 83)	
Hematologic toxicity ≥ 3			
Leukopenia	18 (33.4)	19 (22.9)	0.24
Anemia	3 (5.6)	11 (13.1)	0.25
Thrombocytopenia	6 (11.1)	6 (7.2)	0.54
Esophagitis ≥ 3	10 (18.5)	5 (6.0)	0.027
Esophagitis ≥ 2	21 (38.9)	14 (16.9)	< 0.05
Pneumonitis ≥ 3	4 (7.4)	10 (12.0)	0.40

ENI, elective nodal irradiation; IFI, involved field irradiation

## Discussion

Radiotherapy combined with chemotherapy is the main treatment modality for EC patients with unresectable disease or those who refuse surgery; however, the appropriate irradiation volume remains controversial. There have been reports in the literature that IFI can provide similar OS and LRC with a smaller volume and lower toxicities when compared with ENI. Elderly patients characterized as having poor physiologic status and suffering from comorbidities are underrepresented in clinical trials; therefore, high-grade evidence to guide the treatment of these subgroup is needed. In our retrospective study comparing ENI with IFI for elderly patients (≥ 70 years) with ESCC, the results revealed that the survival of patients with IFI was at least not inferior to that with ENI, and IFI reduced the rate of RE.

Since the final results of RTOG 85–01 were reported, concurrent chemoradiotherapy has been the standard treatment for inoperable EC. However, the volume of radiation therapy is still controversial. In a retrospective analysis, the results were reported for 102 patients diagnosed with ESCC and treated with ENI [5]. Among those, 20 patients experienced treatment failure, for the first site of failure, 10 patients had local failure, 9 patients had distant metastases, and only 1 patient experienced elective nodal failure. Yamashita et al. reported on 126 ESCC patients who received extended ENI with a dose of 50–50.4 Gy; both local failure and distant metastases occurred in 20 (16%) patients without elective nodal failure in any patients [6]. Even though ENI may prevent elective nodal failure or improve local control, it is not clear whether ENI improves OS. The latest National Comprehensive Cancer Network guidelines recommend that the CTV include coverage of elective nodal regions. However, excessive exposure of the irradiated areas would incur increased treatment toxicities. In the RTOG 85–01 trial, 10% of patients experienced life-threatening toxic effects because of combined therapy [4]. In addition, a substantial proportion of patients treated with ENI, especially those who received combined therapy, experienced acute grade ≥ 3 hematological and non-hematological adverse events [6, 9]. Therefore, oncologists should pay greater attention to patients’ safety, and the target of radiotherapy should be considered with caution, particularly in the elderly population.

Recently, IFI has been widely evaluated for the treatment of patients with EC in the clinic [10–16]. In a retrospective analysis, 145 patients with EC, among whom 75% had staged III—IVA disease, were irradiated using IFI with concurrent chemotherapy. The MST and 2-year OS rate were 15 months and 37%, respectively, compared to 16.9 months and 38% observed in the RTOG 85–01 trial, it should be noted that enrolled patients in the latter trial had stage I—III disease and the irradiation modality was ENI [16]. The results indicated that IFI was not inferior to ENI in terms of OS. In a prospectively randomized trial, 102 patients with cervical or upper-thoracic ESCC were treated with concurrent chemoradiotherapy and randomized into the ENI or IFI group; the MST was 33.7 months with IFI vs. 32.7 months with ENI, and there was no significant difference in the 3-year survival rate between the two groups (41.3%, ENI vs. 32.0%, IFI; *p* = 0.58) [11]. Another study has reported that the 3-year OS rate with IFI was even higher than that obtained with ENI (49%, IFI vs. 47%, ENI; *p* = 0.741) [12].

Previous studies indicated that the MST was approximately 9 to 15 months in elderly patients undergoing radiotherapy with or without chemotherapy [17–21]. In a study by Zhang et al., 128 elderly patients (aged ≥ 65 years) with ESCC treated with IFI were analyzed, and the results showed that the median OS and the 3-year OS were 16.0 months and 33.2% [22]. The data, compared with our study, showed a slight improvement in survival. A potential reason for this difference might be the higher proportion of elderly patients in our study compared to that study. In another study, including 58 patients aged ≥ 75 years with adenocarcinoma of the lower esophagus or gastroesophageal junction who were treated with IFI, the MST and 3-year OS rate were 14.5 months and 15.5%, respectively, which were comparable to our data [23]. Another advantage of IFI has been found to be decreased toxicities. RTOG trials indicated that 76% of ENI patients treated with a dose of 64.8 Gy and combined therapy experienced grade ≥ 3 acute toxicities, and 10% of these toxicities were life-threatening [4, 9]. In comparison, Liu et al., reported that only 2 and 5 patients in the IFI group experienced grade ≥ 3 acute RP and RE, respectively [12]. In a recent study, reporting on 63 patients who received IFI with a tumor dose of 50–50.4 Gy and concomitant chemotherapy, the rates of grade ≥ 3 acute esophagitis and leukopenia were 10% and 62%, respectively [15].

Regarding the prognostic roles of clinical factors in elderly patients with ESCC, a consensus has not been reached. In our present study, T stage was found to be likely to affect OS on both univariate analysis (*p* = 0.033) and multivariate analysis (*p* = 0.037). On the other hand, a previous study suggested that involved lymph nodes were the best predictors of survival, while T staging was less predictive for esophageal adenocarcinoma [23]. At present, it seems that TNM staging is still the main factor considered in the prognosis of patients, regardless of whether lymph nodes positive or not associated with prognosis has not been observed.

Few data have been reported on radiation-related toxicities in the elderly. Two studies with small sample sizes address this point. One is a Japanese study including 22 elderly patients who were treated with concurrent chemotherapy consisting of local-field irradiation or prophylactic nodal irradiation; the results indicated that grade ≥ 2 hematological toxicities were less frequent in local-field patients than in the remaining patients (30% vs. 92%, *p* = 0.006) [18]. The other study by Rochigneux et al., evaluated the efficacy of radio(chemo)therapy in elderly patients with IFI, and found that 31% patients (18/58) experienced grade 3–4 side effects, and only 4 patients had grade 1–2 mucositis [24]. In a recent study that included 116 elderly patients (≥ 70 years), in which the treatment modalities were ENI with or without concomitant or sequential chemotherapy, the results were similar to our data regarding the MST (17.9 months vs. 17 months) and the rate of acute grade ≥ 3 esophagitis (17.2% vs. 18.5%) [25]. In our study, only 6% of patients in the IFI group experienced grade ≥ 3 acute esophagitis compared to 18.5% in the ENI group (*p* = 0.027). Further analysis on grade ≥ 2 acute RE also showed a statistical difference between the ENI and IFI groups (38.9% vs. 16.9%; *p* < 0.05). This result was due to the larger volume of the esophagus exposed to irradiation with ENI. Nevertheless, the incidence of the toxicity in 38.9% of patients is still acceptable because grade 2 RE is often well tolerated. Related studies regarding ENI and IFI are summarized in Table 4.

**Table 4:** Studies regarding ENI and/or IFI in esophageal cancer.

Radiation modality	Authors	N	Median age (range)	Stage	OS (%)	MST	Acute grade ≥3 toxicities (%)
ENI	Cooper et al. [4]	61	NR	I-III	30 (3-yr)	14.1	NR
	Minsky et al. [9]	109	64 (37–81)	I-III	40 (2-yr)	18.1	76
	Onozawa et al. [5]	102	64 (39–75)	I-IVB	43 (3-yr)	41	NR
	Yamashita et al. [6]	126	67 (42–75)	I-IVB	43 (3-y)	28.5	26*
	Ma et al. [11]	51	62 (39–74)	I-III	41.3 (3-y)	32.7	64.7^†^
	Kato et al. [7]	51	64 (42–70)	II-III	63.8 (3-y)	NR	35*
	Liu et al. [12]	70	58 (37–90)	I-IV	47 (3-y)	27	6*
	Li et al. [25]	116	76 (70–90)	I-IV	23.2 (3-y)	17.9	17.2*
IFI	Button et al. [16]	145	65.4 (41–83)	I-IVA	37 (2-y)	15	NR
	Zhao et al. [10]	53	64 (44–84)	I-III	41 (3-y)	30	9
	Kawaguchi et al. [13]	68	64 (43–84)	I	76 (3-y)	NR	NR
	Ma et al. [11]	51	62 (39–74)	I-III	32 (3-y)	33.7	27.4*
	Liu et al. [12]	99	62 (37–90)	I-IV	49 (3-y)	27	6*
	Zhang et al. [14]	80	63 (42–74)	I-III	18.8 (3-y)	14.4	NR
	Yamashita et al. [15]	63	67.5 (47–84)	I-IV	51.1 (3-y)	38.4	10*
	Zhang et al. [22]	128	72 (65–89)	I-IV	24.1 (3-y)	16	4.6*
	Rochigneux et al. [24]	58	77.8 (75–87)	I-III	15.5 (3-y)	14.5	31

ENI, elective nodal irradiation; IFI, involved field irradiation; N, numbers of patients; OS, overall survival; MST, median survival time; NR, not reported

* Hematologic toxicity including infection

^†^ Esophagitis

RP is another treatment-related toxicity. Many factors such as irradiation dose and volume, baseline pulmonary function, medical comorbidities and tumor location could influence the incidence of RP. In our study, 10 patients in the IFI group and 2 patients in the ENI group died of RP. The total lung V20 (volume of whole lung receiving 20 Gy) that could be evaluated on the dose volume histogram (DVH) was the major index. According to analysis of the DVH, we found that the proportion of patients with a V20 > 28% in the ENI group was similar to that in the IFI group (5.6% vs. 4.8%). However, the 10 patients who suffered from RP in the IFI group had different degrees of impaired baseline pulmonary function. Of the 10 patients with RP, 3 patients experienced grade ≥ 3 RE. Therefore, it is reasonable to conclude that the primary pulmonary function of elderly patients rather than the influence of the choice of radiotherapy field was the main risk factor of RP.

As our study is a retrospective analysis, there are several inevitable limitations. First, the baseline characteristic and irradiation techniques were not balanced completely between the ENI and IFI groups owing to the moderate number of patients, and thus some bias in the results could not be avoided. Secondly, most patients did not undergo endoscopic ultrasonography; as a result, we could not offer an accurate stage from T1 to T3 through CT examination; however, most patients were at stage T2 or T3 at the time of diagnosis in our cancer center. Therefore, the imprecise stage would not be likely to profoundly impact our conclusion.

## Conclusions

For elderly patients with inoperable EC, the goal of treatment should be to prolong survival as much as possible without reducing the quality of life. The present retrospective study showed that IFI, with a lower incidence of acute RE than ENI, was a feasible treatment strategy and was at least not inferior to ENI in regards to survival in elderly patients. Further large-scale and randomized clinical investigations are needed to affirm the feasibility of IFI for this specific population.

## Supporting Information

S1 File Data of patients with esophageal squamous cell cancer. (XLSX)
