# Correction: Feasibility of Elective Nodal Irradiation (ENI) and Involved Field Irradiation (IFI) in Radiotherapy for the Elderly Patients (Aged ≥ 70 Years) with Esophageal Squamous Cell Cancer: A Retrospective Analysis from a Single Institute

**DOI:** 10.1371/journal.pone.0147453

**Published:** 2016-01-25

**Authors:** Wang Jing, Hui Zhu, Hongbo Guo, Yan Zhang, Fang Shi, Anqin Han, Minghuan Li, Li Kong, Jinming Yu

There is an error in Table 2. Subheadings are missing from the first column. Please see the corrected Table 2 here.

**Table 2:** Univariate and multivariate analysis of the effect of prognostic factors on OS in patients with esophageal cancer.

Factors	Univariate analysis					Multivariate analysis		
	1-y OS (%)	3-y OS (%)	5-y OS (%)	χ2	*p*	HR	95% CI	*p*
**Sex**								
Male	55.0	20.8	12.5	1.467	0.226			
Female	73.7	26.9	0					
**Tumor stage**								
T1-3	64.7	28.4	7.6	4.529	0.033	1.60	1.028–2.495	0.037
T4	57.1	0	0					
**LN status**								
N0	60.6	32.8	10.9	1.245	0.264			
N+	63.5	20.2	4.7					
**Location**								
Cervical	90.9	23.4	11.7	2.252	0.133			
Thoracic	60.3	23.2	5.1					
**Tumor length (cm)**								
≤ 5	64.9	31.7	5.6	2.209	0.154			
> 5	60.3	16.0	5.7					
**RT dose (Gy)**								
≥ 60	65.7	27.7	5.3	0.293	0.588			
< 60	60.0	19.0	6.3					
**KPS score**								
≥ 80	60.0	19.0	6.3	0.293	0.588			
< 80	65.7	27.7	5.3					
**Smoking status**								
Yes	69.4	22.8	3.5	0.066	0.797			
No	55.4	22.9	9.2					
**Treatment strategies**								
RT alone	63.9	21.2	4.7	0.422	0.936			
CCRT	57.1	32.1	6.9					
C+RT	64.0	17.5	8.8					
RT+C	80.0	0	0					
**Irradiation field**								
ENI	68.5	26.4	10.3	1.319	0.251			
IFI	59.0	21.7	0					
